# Supplementary material for: Variance Polygenic Scores (vPGS) as a Tool for Studying Gene-Environment Interactions Associated With Refractive Error
Source: Invest Ophthalmol Vis Sci. 2026 Sep 2;67(11):6. doi: 10.1167/iovs.67.11.6 (PMC13549405; doi:10.1167/iovs.67.11.6)
Supplement: Supplementary file 1 [file iovs-67-11-6_s001.pdf]

Variance polygenic scores (vPGS) as a tool for studying gene-environment interactions associated with refractive error

Supplementary Information

Content

Supplement Note S1. vGWAS analysis details. 2

Supplement Note S2. Derivation of AOSW-inferred SER phenotype. 3

Supplement Note S3. vGWAS effect sizes for vPGS construction. 4

Supplementary Table S1. Performance of SCAMPI vPGS models in the *tuning dataset*......5

Supplementary Table S2. Performance of CQR vPGS models in the *tuning dataset*. 6

Supplementary Table S3. Performance of LT vPGS models in the *tuning dataset*. 8

Supplementary Table S4. Pair-wise comparisons among six vPGS models in the *validation dataset*. 9

Supplementary Table S5. Interaction between mPGS and vPGS and EduAge in the UKB *validation dataset*. 10

Supplementary Table S6. Interaction between mPGS and vPGS and Time Reading in the ALSPAC cohort. 11

Supplementary Table S7. Interaction between mPGS and vPGS and Time Outdoors in the ALSPAC cohort. 12

Supplementary Figure S1. P+T model selection in the *tuning dataset*. 13

Supplementary Figure S2. LDgrid2-grid model selection in the *tuning dataset*. 14

Supplementary Figure S3. Pair-wise comparisons across six vPGS models in the *validation dataset*. 15

References 16

Supplement Note S1. vGWAS analysis details.

As described previously[1], we applied three methods to conduct vGWAS analyses: SCAMPI (Scalable Cauchy Aggregate test using Multiple Phenotypes to test Interactions),[2] conditional quantile regression (CQR),[3] and Levene's test (LT).[4] Based on SCAMPI's requirement to have multiple phenotypes as input, we selected an analysis sample of n = 77,880 UK Biobank participants with information available for both SER and AOSW (Figure 1; *training dataset*). All participants were unrelated, had European genetic ancestry, and did not have a history of past surgical treatment likely to affect SER (see Methods).

The SER and AOSW phenotypes were each regressed on the covariates, age, age^2, sex, genotyping array, and PC1-10. Phenotypes were not transformed to a normal distribution. Approximately 1 million HapMap3 variants (https://doi.org/10.5281/zenodo.7773502) were included in each vGWAS analysis. The R package SCAMPI (https://github.com/epstein-software/SCAMPI) was run using the default settings for the combined residual SER and residual AOSW phenotypes. SCAMPI outputs several p-values: here, we used the p-value for the SER phenotype. CQR was performed for the residual SER phenotype, using the rq function from the R package quantreg version 6.1, at nine quantile values (0.1, 0.2, 0.3, … 0.9). SNP regression coefficients (β_{SNP}) at the nine quantiles (τ) were fit with an inverse variance-weighted restricted maximum likelihood meta-regression model that included an intercept term, a linear term ($\beta_{SNP} \times \tau$) and a quadratic term ($\beta_{SNP} \times \tau^2$) using the rma function from the R package metafor version 4.8-0. Here, we used the CQR meta-regression quadratic term to build the vPGS. To maintain consistency in the sign of the CQR-based vPGS with that of the SCAMP-based vPGS and the LT-based vPGS, we reversed the sign of the CQR-based vPGS so that higher vPGS values indicated higher variance of refractive error. Levene's median test was performed with OSCA (--vqtl option),[4] using the residual SER phenotype. OSCA outputs both a beta coefficient and a p-value for Levene's test.

Supplement Note S2. Derivation of AOSW-inferred SER phenotype.

The AOSW-inferred phenotype has been used in previous studies.[5,6] Here, the *training dataset* (n = 77,880), which contained information about participants' SER and AOSW, was used to train a predictive model for "AOSW-inferred SER". Five-fold cross-validation was used for optimizing the parameters of predictors in the following equation:

$$\mathrm{AOSW-inferred\ SER} \sim \mathrm{sex} + \mathrm{poly}(\mathrm{AOSW}, \mathrm{n}) + \mathrm{poly}(\mathrm{age}, \mathrm{m})$$

Where, poly(a, b) is a R function to generate orthogonal polynomials up to order b for predictor a. The parameter, b was tested across the range m = 1–6 for age, and n = 1–15 for AOSW. Based on the Akaike Information Criterion (AIC) of the 5-fold cross-validation analysis, optimal settings were, n = 13 and m = 6. This model was used to predict the AOSW-inferred SER of participants in the *tuning dataset* (n = 221,516).

Supplement Note S3. vGWAS effect sizes for vPGS construction.

Beta coefficients quantifying SNP effect sizes are required to construct a PGS or vPGS. SNP effect sizes for the CQR vGWAS meta-regression quadratic term were used as the effect size for building a CQR vPGS. The beta coefficients from the OSCA Levene's median test were used as the effect size for LT vPGS construction. SCAMPI outputs p-values but not beta coefficients. Therefore, the p-values form SCAMPI were transformed to Z-values (formula 1), where Φ is the standard normal cumulative distribution function. The sign of the Z-value for each SNP was borrowed from the Levene's test vGWAS summary statistics. The standard error (SE) of the effect size of each variant was calculated (formula 2), which required the sample size (N) and minor allele frequencies (MAF) of each variant. Finally, the SNP effect size (β) was calculated (formula 3).

$$|Z| = \Phi^{-1}\left(1 - \frac{P}{2}\right) \quad (1)$$

$$SE = \frac{1}{\sqrt{2N \times MAF \times (1 - MAF)}} \quad (2)$$

$$\beta = Z \times SE \quad (3)$$

Supplementary Table S1. Performance of SCAMPI vPGS models in the *tuning dataset*.

Method	Tuning parameters	Diff (95% CI)	Spearman Correlation (95% CI)
P+T	P<5.00E-08	0.18 (0.13, 0.22)	1.00 (0.87, 1.00)
P+T	P<1.00E-07	0.20 (0.15, 0.24)	0.99 (0.90, 1.00)
P+T	P<1.00E-06	0.21 (0.17, 0.26)	0.96 (0.89, 1.00)
P+T	P<1.00E-05	0.18 (0.14, 0.23)	0.98 (0.85, 0.99)
P+T	P<1.00E-04	0.23 (0.18, 0.27)	0.99 (0.89, 1.00)
P+T	P<1.00E-03	0.23 (0.18, 0.27)	0.98 (0.83, 0.99)
P+T	P<1.00E-02	0.20 (0.15, 0.24)	0.99 (0.84, 0.99)
P+T	P<5.00E-02	0.18 (0.14, 0.23)	1.00 (0.90, 1.00)
P+T	P<1.00E-01	0.20 (0.16, 0.24)	0.96 (0.89, 0.99)
P+T	P<1.00	0.20 (0.16, 0.25)	0.99 (0.89, 1.00)
LDpred2-inf	-	0.29 (0.24, 0.33)	1.00 (0.90, 1.00)
LDpred2-grid	P=0.001; H^2=0.05; S=FALSE	0.13 (0.08, 0.17)	0.94 (0.71, 0.95)
LDpred2-grid	P=0.01; H^2=0.05; S=FALSE	0.34 (0.29, 0.38)	1.00 (0.94, 1.00)
LDpred2-grid	P=0.1 H^2=0.05; S=FALSE	0.30 (0.25, 0.34)	1.00 (0.93, 1.00)
LDpred2-grid	P=1; H^2=0.05; S=FALSE	0.30 (0.26, 0.34)	1.00 (0.92, 1.00)
LDpred2-grid	P=0.001; H^2=0.1; S=FALSE	0.08 (0.04, 0.12)	0.94 (0.56, 0.95)
LDpred2-grid	P=0.01; H^2=0.1; S=FALSE	0.30 (0.26, 0.34)	1.00 (0.95, 1.00)
LDpred2-grid	P=0.1 H^2=0.1; S=FALSE	0.32 (0.27, 0.36)	0.99 (0.94, 1.00)
LDpred2-grid	P=1; H^2=0.1; S=FALSE	0.29 (0.25, 0.33)	0.95 (0.89, 1.00)
LDpred2-grid	P=0.01; H^2=0.15; S=FALSE	0.28 (0.24, 0.33)	1.00 (0.93, 1.00)
LDpred2-grid	P=0.1; H^2=0.15; S=FALSE	0.30 (0.25, 0.34)	0.99 (0.93, 1.00)
LDpred2-grid	P=1 H^2=0.15; S=FALSE	0.29 (0.24, 0.33)	0.99 (0.91, 1.00)
LDpred2-grid	P=0.01; H^2=0.20; S=FALSE	0.27 (0.23, 0.32)	0.98 (0.89, 1.00)
LDpred2-grid	P=0.1; H^2=0.20; S=FALSE	0.29 (0.24, 0.33)	0.99 (0.92, 1.00)
LDpred2-grid	P=1 H^2=0.20; S=FALSE	0.29 (0.24, 0.33)	0.99 (0.89, 0.99)
LDpred2-grid	P=0.001; H^2=0.05; S=TRUE	0.10 (0.06, 0.15)	0.87 (0.61, 0.96)
LDpred2-grid	P=0.01; H^2=0.05; S=TRUE	0.33 (0.29, 0.38)	1.00 (0.93, 1.00)
LDpred2-grid	P=0.1 H^2=0.05; S=TRUE	0.30 (0.26, 0.34)	0.99 (0.94, 1.00)
LDpred2-grid	P=1; H^2=0.05; S=TRUE	0.30 (0.25, 0.34)	1.00 (0.94, 1.00)
LDpred2-grid	P=0.001; H^2=0.10; S=TRUE	0.10 (0.06, 0.15)	0.94 (0.61, 0.95)
LDpred2-grid	P=0.01; H^2=0.10; S=TRUE	0.29 (0.24, 0.34)	0.99 (0.92, 1.00)
LDpred2-grid	P=0.1 H^2=0.10; S=TRUE	0.30 (0.25, 0.34)	0.98 (0.92, 1.00)
LDpred2-grid	P=1; H^2=0.10; S=TRUE	0.29 (0.25, 0.33)	0.99 (0.90, 1.00)
LDpred2-grid	P=0.01; H^2=0.15; S=TRUE	0.26 (0.22, 0.30)	1.00 (0.93, 1.00)
LDpred2-grid	P=0.1 H^2=0.15; S=TRUE	0.30 (0.26, 0.34)	1.00 (0.95, 1.00)
LDpred2-grid	P=1; H^2=0.15; S=TRUE	0.28 (0.23, 0.32)	1.00 (0.90, 1.00)
LDpred2-grid	P=0.01; H^2=0.20; S=TRUE	0.23 (0.19, 0.28)	0.99 (0.89, 1.00)
LDpred2-grid	P=0.1 H^2=0.20; S=TRUE	0.30 (0.26, 0.35)	0.99 (0.94, 1.00)
LDpred2-grid	P=1; H^2=0.20; S=TRUE	0.28 (0.24, 0.32)	1.00 (0.92, 1.00)

P=P threshold; H^2=heritability; S=sparsity.

Supplementary Table S2. Performance of CQR vPGS models in the *tuning dataset*.

Method	Tuning parameters	Diff (95% CI)	Spearman Correlation (95% CI)
P+T	P<5.00E-08	0.16 (0.11, 0.20)	0.96 (0.85, 0.96)
P+T	P<1.00E-07	0.16 (0.11, 0.20)	0.96 (0.85, 0.96)
P+T	P<1.00E-06	0.15 (0.10, 0.19)	0.99 (0.83, 0.99)
P+T	P<1.00E-05	0.18 (0.14, 0.23)	0.96 (0.82, 0.99)
P+T	P<1.00E-04	0.18 (0.13, 0.22)	0.90 (0.77, 0.99)
P+T	P<1.00E-03	0.24 (0.20, 0.29)	0.98 (0.87, 0.99)
P+T	P<1.00E-02	0.22 (0.17, 0.26)	1.00 (0.88, 1.00)
P+T	P<5.00E-02	0.24 (0.20, 0.28)	0.96 (0.90, 1.00)
P+T	P<1.00E-01	0.27 (0.22, 0.31)	1.00 (0.92, 1.00)
P+T	P<1.00	0.26 (0.22, 0.30)	0.99 (0.93, 1.00)
LDpred2-inf	-	0.283 (0.24, 0.33)	0.99 (0.94, 1.00)
LDpred2-grid	P=0.0001; H^2=0.05; S=FALSE	0.24 (0.2, 0.29)	1.00 (0.89, 1.00)
LDpred2-grid	P=0.001; H^2=0.05; S=FALSE	0.29 (0.25, 0.33)	0.99 (0.93, 0.99)
LDpred2-grid	P=0.01; H^2=0.05; S=FALSE	0.34 (0.29, 0.38)	0.99 (0.96, 1.00)
LDpred2-grid	P=0.1 H^2=0.05; S=FALSE	0.30 (0.26, 0.35)	0.99 (0.93, 1.00)
LDpred2-grid	P=1; H^2=0.05; S=FALSE	0.29 (0.24, 0.33)	0.99 (0.94, 1.00)
LDpred2-grid	P=0.0001; H^2=0.1; S=FALSE	0.22 (0.17, 0.26)	0.99 (0.89, 1.00)
LDpred2-grid	P=0.001; H^2=0.1; S=FALSE	0.29 (0.24, 0.33)	0.99 (0.93, 1.00)
LDpred2-grid	P=0.01; H^2=0.1; S=FALSE	0.33 (0.28, 0.37)	0.98 (0.94, 0.99)
LDpred2-grid	P=0.1 H^2=0.1; S=FALSE	0.30 (0.26, 0.35)	1.00 (0.95, 1.00)
LDpred2-grid	P=1; H^2=0.1; S=FALSE	0.28 (0.24, 0.33)	0.99 (0.94, 1.00)
LDpred2-grid	P=0.0001; H^2=0.15; S=FALSE	0.22 (0.18, 0.26)	0.99 (0.90, 1.00)
LDpred2-grid	P=0.001; H^2=0.15; S=FALSE	0.27 (0.23, 0.32)	0.96 (0.91, 0.99)
LDpred2-grid	P=0.01; H^2=0.15; S=FALSE	0.33 (0.29, 0.38)	0.99 (0.95, 1.00)
LDpred2-grid	P=0.1; H^2=0.15; S=FALSE	0.31 (0.26, 0.35)	0.99 (0.93, 1.00)
LDpred2-grid	P=1 H^2=0.15; S=FALSE	0.29 (0.25, 0.33)	0.99 (0.93, 0.99)
LDpred2-grid	P=0.0001; H^2=0.20; S=FALSE	0.21 (0.17, 0.26)	0.96 (0.89, 0.99)
LDpred2-grid	P=0.001; H^2=0.20; S=FALSE	0.27 (0.22, 0.31)	0.96 (0.93, 1.00)
LDpred2-grid	P=0.01; H^2=0.20; S=FALSE	0.32 (0.28, 0.37)	0.99 (0.93, 1.00)
LDpred2-grid	P=0.1; H^2=0.20; S=FALSE	0.32 (0.28, 0.37)	0.99 (0.94, 1.00)
LDpred2-grid	P=1 H^2=0.20; S=FALSE	0.30 (0.25, 0.34)	0.96 (0.95, 1.00)
LDpred2-grid	P=0.0001; H^2=0.05; S=TRUE	0.22 (0.18, 0.26)	0.99 (0.90, 1.00)
LDpred2-grid	P=0.001; H^2=0.05; S=TRUE	0.27 (0.22, 0.32)	0.98 (0.92, 0.99)
LDpred2-grid	P=0.01; H^2=0.05; S=TRUE	0.33 (0.28, 0.38)	0.98 (0.94, 1.00)
LDpred2-grid	P=0.1 H^2=0.05; S=TRUE	0.30 (0.26, 0.34)	0.99 (0.93, 1.00)
LDpred2-grid	P=1; H^2=0.05; S=TRUE	0.29 (0.25, 0.34)	0.99 (0.94, 1.00)
LDpred2-grid	P=0.0001; H^2=0.10; S=TRUE	0.23 (0.18, 0.27)	0.99 (0.88, 0.99)
LDpred2-grid	P=0.001; H^2=0.10; S=TRUE	0.27 (0.23, 0.31)	0.98 (0.92, 1.00)
LDpred2-grid	P=0.01; H^2=0.10; S=TRUE	0.34 (0.29, 0.38)	0.98 (0.95, 1.00)
LDpred2-grid	P=0.1 H^2=0.10; S=TRUE	0.31 (0.26, 0.35)	0.99 (0.94, 1.00)

LDpred2-grid	P=1; H^2=0.10; S=TRUE	0.29 (0.25, 0.34)	0.99 (0.94, 1.00)
LDpred2-grid	P=0.0001; H^2=0.15; S=TRUE	0.21 (0.16, 0.25)	0.98 (0.89, 1.00)
LDpred2-grid	P=0.001; H^2=0.15; S=TRUE	0.27 (0.23, 0.31)	1.00 (0.93, 1.00)
LDpred2-grid	P=0.01; H^2=0.15; S=TRUE	0.33 (0.29, 0.37)	0.98 (0.95, 1.00)
LDpred2-grid	P=0.1 H^2=0.15; S=TRUE	0.31 (0.26, 0.35)	0.96 (0.94, 1.00)
LDpred2-grid	P=1; H^2=0.15; S=TRUE	0.29 (0.24, 0.34)	1.00 (0.94, 1.00)
LDpred2-grid	P=0.0001; H^2=0.20; S=TRUE	0.22 (0.18, 0.26)	0.95 (0.88, 0.99)
LDpred2-grid	P=0.001; H^2=0.20; S=TRUE	0.26 (0.22, 0.31)	0.96 (0.90, 0.99)
LDpred2-grid	P=0.01; H^2=0.20; S=TRUE	0.33 (0.28, 0.37)	1.00 (0.95, 1.00)
LDpred2-grid	P=0.1 H^2=0.20; S=TRUE	0.32 (0.27, 0.36)	0.99 (0.95, 1.00)
LDpred2-grid	P=1; H^2=0.20; S=TRUE	0.32 (0.27, 0.37)	0.99 (0.95, 1.00)

P=P threshold for LDpred2-grid model; H^2=heritability; S=sparsity.

Supplementary Table S3. Performance of LT vPGS models in the *tuning dataset*.

Method	Tuning parameters	Diff (95% CI)	Spearman Correlation (95% CI)
P+T	P<5.00E-08	0.24 (0.19, 0.28)	0.93 (0.81, 0.99)
P+T	P<1.00E-07	0.24 (0.19, 0.28)	0.93 (0.83, 0.99)
P+T	P<1.00E-06	0.23 (0.18, 0.27)	0.99 (0.85, 1.00)
P+T	P<1.00E-05	0.20 (0.15, 0.24)	0.98 (0.87, 1.00)
P+T	P<1.00E-04	0.15 (0.11, 0.20)	0.94 (0.84, 0.99)
P+T	P<1.00E-03	0.17 (0.13, 0.22)	0.96 (0.82, 0.99)
P+T	P<1.00E-02	0.22 (0.18, 0.27)	0.98 (0.84, 0.99)
P+T	P<5.00E-02	0.24 (0.20, 0.29)	0.99 (0.83, 1.00)
P+T	P<1.00E-01	0.24 (0.20, 0.29)	0.99 (0.87, 1.00)
P+T	P<1.00	0.24 (0.20, 0.29)	0.99 (0.81, 0.99)
LDpred2-infinitesimal	-	0.32 (0.27, 0.36)	0.99 (0.94, 1.00)
LDpred2-grid	P=0.001; H^2=0.05; S=FALSE	0.16 (0.11, 0.20)	0.99 (0.61, 0.98)
LDpred2-grid	P=0.01; H^2=0.05; S=FALSE	0.35 (0.31, 0.40)	0.98 (0.95, 1.00)
LDpred2-grid	P=0.1 H^2=0.05; S=FALSE	0.32 (0.27, 0.37)	0.98 (0.92, 1.00)
LDpred2-grid	P=1; H^2=0.05; S=FALSE	0.31 (0.27, 0.36)	0.99 (0.92, 1.00)
LDpred2-grid	P=0.001; H^2=0.1; S=FALSE	0.11 (0.07, 0.16)	0.96 (0.68, 0.98)
LDpred2-grid	P=0.01; H^2=0.1; S=FALSE	0.33 (0.28, 0.37)	1.00 (0.96, 1.00)
LDpred2-grid	P=0.1 H^2=0.1; S=FALSE	0.34 (0.29, 0.38)	0.98 (0.92, 1.00)
LDpred2-grid	P=1; H^2=0.1; S=FALSE	0.33 (0.28, 0.37)	0.99 (0.94, 1.00)
LDpred2-grid	P=0.001; H^2=0.15; S=FALSE	0.09 (0.042, 0.13)	0.90 (0.61, 0.95)
LDpred2-grid	P=0.01; H^2=0.15; S=FALSE	0.33 (0.28, 0.37)	1.00 (0.93, 1.00)
LDpred2-grid	P=0.1; H^2=0.15; S=FALSE	0.33 (0.29, 0.38)	0.99 (0.93, 1.00)
LDpred2-grid	P=1 H^2=0.15; S=FALSE	0.32 (0.28, 0.37)	0.99 (0.90, 1.00)
LDpred2-grid	P=0.01; H^2=0.20; S=FALSE	0.28 (0.23, 0.32)	0.99 (0.93, 1.00)
LDpred2-grid	P=0.1; H^2=0.20; S=FALSE	0.33 (0.29, 0.38)	0.98 (0.92, 1.00)
LDpred2-grid	P=1 H^2=0.20; S=FALSE	0.33 (0.28, 0.37)	0.98 (0.92, 1.00)
LDpred2-grid	P=0.001; H^2=0.05; S=TRUE	0.14 (0.094, 0.18)	0.93 (0.73, 0.98)
LDpred2-grid	P=0.01; H^2=0.05; S=TRUE	0.36 (0.32, 0.41)	1.00 (0.96, 1.00)
LDpred2-grid	P=0.1 H^2=0.05; S=TRUE	0.33 (0.29, 0.38)	0.98 (0.92, 1.00)
LDpred2-grid	P=1; H^2=0.05; S=TRUE	0.33 (0.28, 0.37)	0.99 (0.93, 1.00)
LDpred2-grid	P=0.001; H^2=0.10; S=TRUE	0.10 (0.057, 0.15)	0.94 (0.70, 0.98)
LDpred2-grid	P=0.01; H^2=0.10; S=TRUE	0.34 (0.29, 0.38)	1.00 (0.92, 1.00)
LDpred2-grid	P=0.1 H^2=0.10; S=TRUE	0.35 (0.3, 0.39)	0.99 (0.93, 1.00)
LDpred2-grid	P=1; H^2=0.10; S=TRUE	0.32 (0.27, 0.36)	0.98 (0.92, 1.00)
LDpred2-grid	P=0.001; H^2=0.15; S=TRUE	0.1 (0.058, 0.14)	0.94 (0.58, 0.96)
LDpred2-grid	P=0.01; H^2=0.15; S=TRUE	0.31 (0.27, 0.36)	0.99 (0.93, 1.00)
LDpred2-grid	P=0.1 H^2=0.15; S=TRUE	0.35 (0.30, 0.39)	0.99 (0.93, 1.00)
LDpred2-grid	P=1; H^2=0.15; S=TRUE	0.33 (0.28, 0.37)	0.99 (0.94, 1.00)
LDpred2-grid	P=0.01; H^2=0.20; S=TRUE	0.28 (0.24, 0.32)	0.99 (0.90, 1.00)
LDpred2-grid	P=0.1 H^2=0.20; S=TRUE	0.34 (0.30, 0.38)	0.99 (0.93, 1.00)
LDpred2-grid	P=1; H^2=0.20; S=TRUE	0.31 (0.27, 0.35)	0.98 (0.91, 1.00)

P=P threshold for LDpred2-grid model; H^2=heritability; S=sparsity

Supplementary Table S4. Pair-wise comparisons among six vPGS models in the *validation dataset*.

Model 1	Model 2	$Diff_{Model\ 2}$ - $Diff_{Model\ 1}$	Spearman $rho_{Model\ 2}$ - Spearman $rho_{Model\ 1}$
SCAMPI (P+T)	CQR.(P+T)	(-0.43, 1.54)	(-0.034, 0.004)
SCAMPI (P+T)	LT.(P+T)	(-1.59, 0.74)	(0.019, 0.054)
SCAMPI (P+T)	SCAMPI.(LDpred2)	(-1.28, 0.26)	(-0.066, -0.039)
SCAMPI (P+T)	CQR.(LDpred2)	(-2.13, -0.20)	(-0.085, -0.053)
SCAMPI (P+T)	LT.(LDpred2)	(-1.08, 0.76)	(-0.059, -0.024)
CQR (P+T)	LT.(P+T)	(-2.11, 0.29)	(0.032, 0.072)
CQR (P+T)	SCAMPI.(LDpred2)	(-1.91, -0.14)	(-0.054, -0.019)
CQR (P+T)	CQR.(LDpred2)	(-2.74, -0.81)	(-0.066, -0.042)
CQR (P+T)	LT.(LDpred2)	(-1.76, 0.27)	(-0.042, -0.0083)
LT (P+T)	SCAMPI.(LDpred2)	(-1.19, 0.98)	(-0.11, -0.071)
LT (P+T)	CQR.(LDpred2)	(-1.83, 0.30)	(-0.12, -0.087)
LT (P+T)	LT.(LDpred2)	(-0.87, 1.39)	(-0.094, -0.06)
SCAMPI (LDpred2)	CQR.(LDpred2)	(-1.54, 0.20)	(-0.029, -0.053)
SCAMPI (LDpred2)	LT.(LDpred2)	(-0.51, 1.18)	(-0.0027, 0.024)
CQR (LDpred2)	LT.(LDpred2)	(0.077, 1.97)	(0.012, 0.043)

Values within brackets are 95% confidence intervals (CIs) of bootstrapping parameters. All differences were derived by subtracting Diff or Spearman correlation in Model 2 from Model 1.

Supplementary Table S5. Interaction between mPGS and vPGS and EduAge in the UKB *validation dataset*.

Terms	Model 1			Model 2			Model 3		
	**BETA**	**SE**	***P***	**BETA**	**SE**	***P***	**BETA**	**SE**	***P***
Intercept	9.55	0.69	4.64E-43	10.17	0.72	1.41E-45	9.56	0.69	3.67E-43
Sex (female)	0.029	0.029	0.32	0.026	0.030	0.39	0.031	0.029	0.30
Age (years)	-0.31	0.025	6.29E-35	-0.33	0.026	1.77E-36	-0.31	0.025	5.60E-35
Squared age (years^2)	0.0031	0.0002	3.48E-41	0.0032	0.0002	4.12E-42	0.0031	0.0002	3.12E-41
Genotyping array	-0.070	0.047	0.14	-0.094	0.049	0.053	-0.066	0.047	0.16
PC1	-0.004	0.009	0.62	-0.0063	0.009	0.50	-0.005	0.009	0.60
PC2	-0.0016	0.009	0.86	-0.0037	0.010	0.70	-0.0014	0.010	0.88
PC3	-0.0026	0.009	0.78	-0.0015	0.010	0.88	-0.0028	0.009	0.76
PC4	-0.0053	0.005	0.29	-0.016	0.005	2.05E-03	-0.0032	0.005	0.53
PC5	0.0060	0.002	0.01	-0.003	0.002	0.21	0.0078	0.002	9.00E-04
PC6	-0.0074	0.009	0.39	-0.0057	0.009	0.51	-0.008	0.009	0.35
PC7	-0.011	0.006	0.051	-0.0092	0.006	0.12	-0.012	0.006	0.044
PC8	-0.00012	0.006	0.98	0.0045	0.006	0.49	-0.0006	0.006	0.92
PC9	-3.19E-05	0.004	0.99	0.0010	0.004	0.79	0.00033	0.004	0.93
PC10	0.0094	0.007	0.18	0.0075	0.007	0.30	0.011	0.007	0.13
**EduAge (years)**	**-0.12**	**0.006**	**5.79E-85**	**-0.12**	**0.006**	**1.64E-82**	**-0.12**	**0.006**	**3.57E-86**
**mPGS (Z-score)**	**0.28**	**0.12**	**1.60E-02**	**-**	**-**	**-**	**0.08**	**0.15**	**0.60**
**mPGS×EduAge**	**0.023**	**0.006**	**2.86E-04**	**-**	**-**	**-**	**0.037**	**0.008**	**9.31E-06**
**vPGS (Z-score)**	**-**	**-**	**-**	**-0.40**	**0.12**	**8.25E-04**	**-0.30**	**0.15**	**0.052**
**vPGS×EduAge**	**-**	**-**	**-**	**-0.002**	**0.006**	**0.76**	**0.021**	**0.008**	**0.011**

Model 1. SER ~ EduAge + mPGS + mPGS × EduAge + covariates.

Model 2. SER ~ EduAge + vPGS + vPGS × EduAge + covariates.

Model 3. SER ~ EduAge + mPGS + mPGS × EduAge + vPGS + vPGS × EduAge + covariates.

Covariates: Sex, age, squared age, PC1-PC10, genotyping array.

Supplementary Table S6. Interaction between mPGS and vPGS and Time Reading in the ALSPAC cohort.

Terms	Model 1			Model 2			Model 3		
	BETA	SE	*P*	BETA	SE	*P*	BETA	SE	*P*
Intercept	47.12	34.30	0.17	50.98	35.22	0.15	46.97	34.31	0.17
Sex (female)	0.032	0.042	0.44	0.031	0.043	0.47	0.033	0.042	0.44
Age (years)	-6.13	4.37	0.16	-6.63	4.49	0.14	-6.11	4.38	0.16
Squared age (years^2)	0.20	0.14	0.15	0.22	0.14	0.13	0.20	0.14	0.16
PC1	-22.26	11.36	0.05	-19.36	11.67	0.10	-22.49	11.37	0.048
PC2	9.23	10.28	0.37	8.85	10.56	0.40	9.22	10.28	0.37
PC3	-8.48	9.23	0.36	-10.83	9.48	0.25	-8.44	9.23	0.36
PC4	-11.70	9.77	0.23	-16.63	10.05	0.098	-11.49	9.79	0.24
PC5	-5.94	9.90	0.55	-3.86	10.16	0.70	-5.86	9.90	0.55
PC6	-19.11	10.31	0.064	-16.57	10.59	0.12	-19.11	10.32	0.064
PC7	0.073	10.03	0.99	5.90	10.29	0.57	-0.016	10.03	0.99
PC8	-6.16	10.55	0.60	-7.41	10.83	0.49	-6.13	10.55	0.56
PC9	12.57	10.06	0.21	14.69	10.33	0.16	12.50	10.06	0.21
PC10	-14.68	10.69	0.17	-16.14	10.98	0.14	-14.75	10.70	0.17
**Time reading (high)**¶	-0.21	0.043	1.29E-06	-0.22	0.044	6.50E-07	-0.21	0.043	1.25E-06
**mPGS (Z-score)**	0.35	0.026	1.40E-39	-	-	-	0.35	0.036	3.89E-23
**mPGS×Reading time**	0.061	0.043	0.15	-	-	-	0.071	0.057	0.21
**vPGS (Z-score)**	-	-	-	-0.23	0.027	9.40E-18	0.0047	0.035	0.89
**vPGS×Reading time**	-	-	-	-0.034	0.044	0.43	0.015	0.057	0.80

Model 1: SER ~ Time reading + mPGS + mPGS × Time reading + covariates.

Model 2: SER ~ Time reading + vPGS + vPGS × Time reading + covariates.

Model 3: SER ~ Time reading + mPGS + mPGS × Time reading + vPGS + vPGS × Time reading + covariates.

Covariates: Sex, age, squared age, PC1-PC10.

¶Time spent reading was assessed when participants were about 8 years old. SER and age were assessed when participants were about 15 years old.

Supplementary Table S7. Interaction between mPGS and vPGS and Time Outdoors in the ALSPAC cohort.

Terms	Model 1			Model 2			Model 3		
	BETA	SE	*P*	BETA	SE	*P*	BETA	SE	*P*
Intercept	46.98	34.40	0.17	49.67	35.34	0.16	46.72	34.42	0.17
Sex (female)	-0.0022	0.042	0.96	-0.0036	0.043	0.93	-0.0021	0.042	0.96
Age (years)	-6.11	4.39	0.16	-6.47	4.51	0.15	-6.08	4.39	0.17
Squared age (years^2)	0.20	0.14	0.16	0.21	0.14	0.14	0.20	0.14	0.16
PC1	-20.43	11.39	0.073	-17.99	11.70	0.12	-20.61	11.40	0.071
PC2	9.66	10.31	0.35	9.07	10.62	0.39	9.56	10.34	0.36
PC3	-9.69	9.25	0.29	-12.01	9.50	0.21	-9.69	9.26	0.30
PC4	-10.85	9.80	0.27	-15.65	10.08	0.12	-10.59	9.82	0.28
PC5	-6.58	9.93	0.51	-4.58	10.20	0.65	-6.59	9.93	0.51
PC6	-19.94	10.34	0.054	-17.18	10.62	0.11	-19.97	10.34	0.054
PC7	-0.51	10.06	0.96	5.41	10.33	0.60	-0.63	10.06	0.95
PC8	-5.57	10.58	0.60	-6.99	10.87	0.52	-5.55	10.58	0.60
PC9	11.2805	10.09	0.26	13.50	10.36	0.19	11.20	10.09	0.27
PC10	-13.67	10.73	0.20	-15.44	11.03	0.16	-13.70	10.74	0.20
**Time outdoors (high)**¶	0.15	0.072	0.043	0.13	0.075	0.079	0.15	0.073	0.041
**mPGS (Z-score)**	0.27	0.064	2.35E-05	-	-	-	0.29	0.089	1.35E-03
**mPGS×Outdoor time**	0.12	0.067	0.073	-	-	-	0.11	0.094	0.23
**vPGS (Z-score)**	-	-	-	-0.19	0.070	6.5E-03	0.024	0.095	0.80
**vPGS×Outdoor time**	-	-	-	-0.064	0.073	0.38	-0.014	0.099	0.89

Model 1: SER ~ Time outdoors + mPGS + mPGS × Time outdoors + covariates.

Model 2: SER ~ Time outdoors + vPGS + vPGS × Time outdoors + covariates.

Model 3: SER ~ Time outdoors + mPGS + mPGS × Time outdoors + vPGS + vPGS × Time outdoors + covariates.

Covariates: Sex, age, squared age, PC1-PC10.

¶Time spent outdoors was assessed when participants were about 8 years old. SER and age were assessed when participants were about 15 years old.

Supplementary Figure S1. P+T model selection in the *tuning dataset*.

vPGS performance quantified using the Diff metric across a range of p-value thresholds, using vGWAS summary statistics from SCAMPI, conditional quantile regression (CQR), or Levene's test (LT).

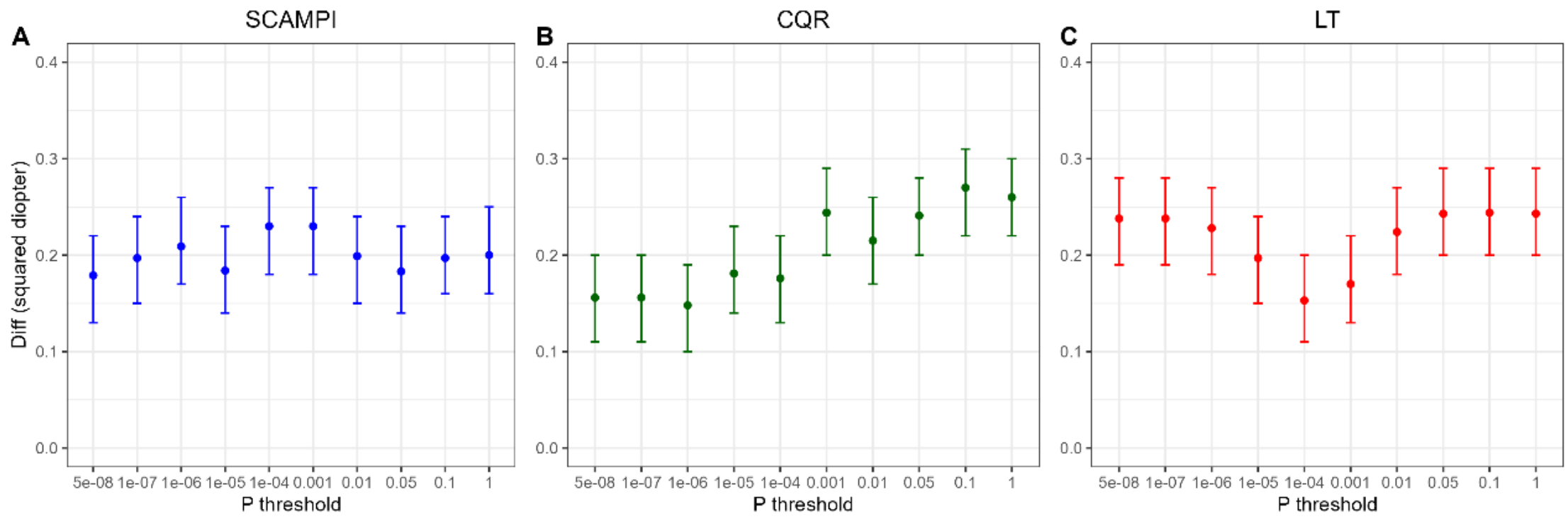

Supplementary Figure S2. LDgrid2-grid model selection in the *tuning dataset*. Diff: the difference between the variance of residual SER in 10th vPGS decile vs. 1st vPGS decile. **A**: vPGS constructed from SCAMPI summary statistics; **B**: vPGS constructed from CQR summary statistics; **C**: vPGS constructed from LT summary statistics. True and False refer to the sparsity parameter of the LDpred2 model. Results were obtained for the trait residuals of AOSW-inferred SER after adjusting for age, age^2, sex, genotyping array, PC1-10, and the optimal conventional PGS (mPGS).

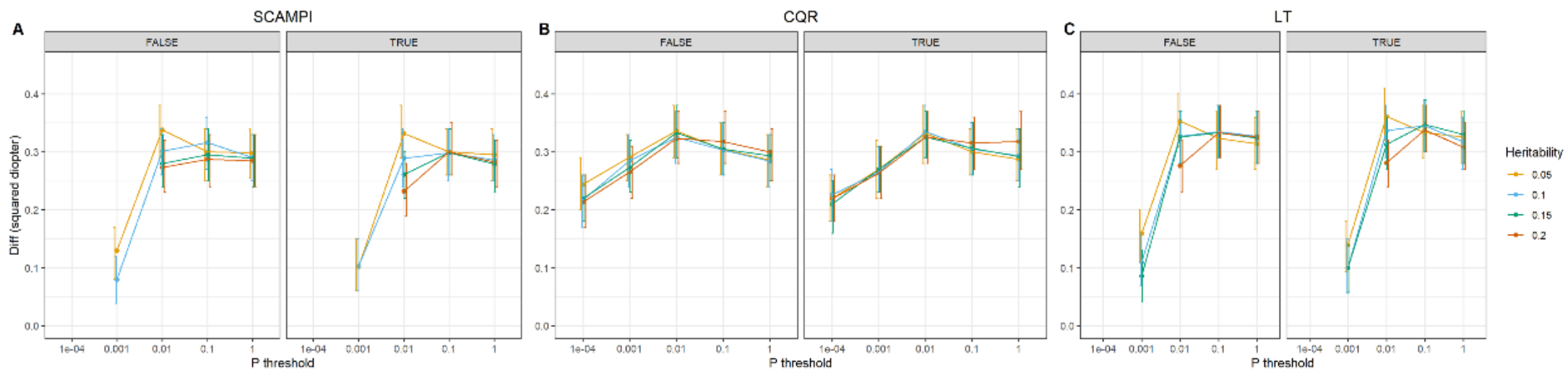

Supplementary Figure S3. Pair-wise comparisons across six vPGS models in the *validation dataset*. The upper triangle of the heatmap demonstrates the difference in the Spearman correlation between two methods, while the lower triangle demonstrates the difference in the Diff value between the two methods. Green, red and blue colored squares indicate that CQR, LT or SCAMPI, respectively, was the vGWAS method that performed significantly better in the pairwise comparison. Hatched and unhatched squares indicate that pruning and thresholding (P+T) or LDpred2, respectively, was the vPGS algorithm that performed significantly better in the pairwise comparison. Full results are shown in Table S4.

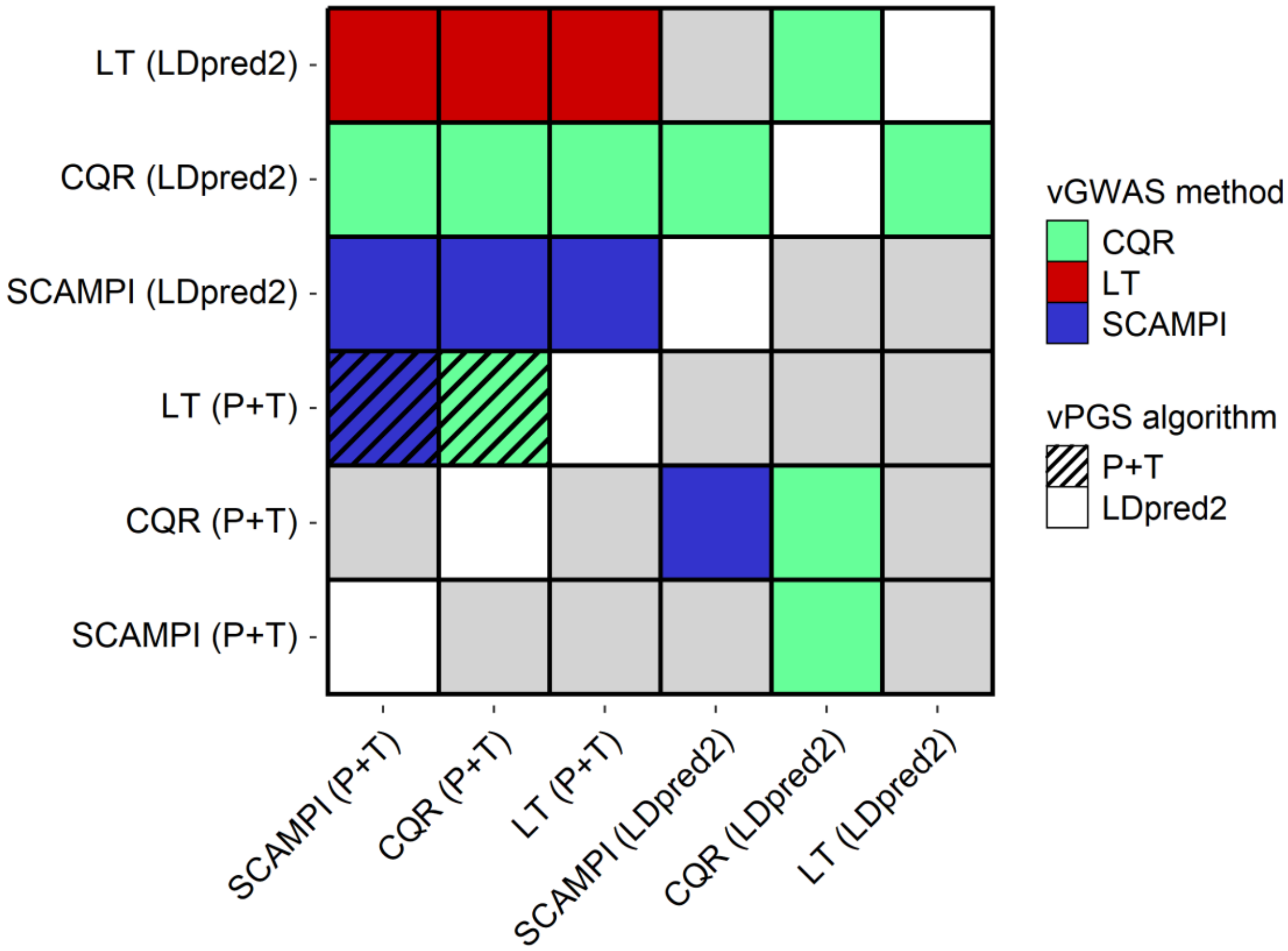

References

1. He X, Terry L, Guggenheim JA, Eye UKB, Vision C. Gene-Environment Interaction Loci Associated with Refractive Error: SCAMPI Analysis. *Ophthalmol Sci.* 2026;6:101219.

2. Bian S, Bass AJ, Liu Y, et al. SCAMPI: A scalable statistical framework for genome-wide interaction testing harnessing cross-trait correlations. *bioRxiv.* 2024.

3. Pozarickij A, Williams C, Hysi PG, Guggenheim JA, U. K. Biobank Eye and Vision Consortium. Quantile regression analysis reveals widespread evidence for gene-environment or gene-gene interactions in myopia development. *Comms Biol.* 2019;2:167.

4. Zhang F, Chen W, Zhu Z, et al. OSCA: a tool for omic-data-based complex trait analysis. *Genome Biol.* 2019;20:107.

5. Ghorbani Mojarrad N, Plotnikov D, Williams C, Guggenheim JA, U.K. Biobank Eye & Vision Consortium. Association Between Polygenic Risk Score and Risk of Myopia. *JAMA Ophthalmol.* 2020;138:7–13.

6. Clark R, Lee SS, Du R, et al. A new polygenic score for refractive error improves detection of children at risk of high myopia but not the prediction of those at risk of myopic macular degeneration. *EBioMed.* 2023;91:104551.
